# Telemonitoring Interventions in COPD Patients: Overview of Systematic Reviews

**DOI:** 10.1155/2020/5040521

**Published:** 2020-01-16

**Authors:** Xuanlin Li, Yang Xie, Hulei Zhao, Hailong Zhang, Xueqing Yu, Jiansheng Li

**Affiliations:** ^1^Co-Construction Collaborative Innovation Center for Chinese Medicine and Respiratory Diseases by Henan & Education Ministry of China, Zhengzhou, Henan 450046, China; ^2^Henan Key Laboratory of Chinese Medicine for Respiratory Disease, Henan University of Chinese Medicine, Zhengzhou, Henan 450046, China; ^3^Department of Respiratory Diseases, The First Affiliated Hospital of Henan University of Chinese Medicine, Zhengzhou, Henan 450000, China

## Abstract

**Objective:**

The role of telemonitoring interventions (TIs) for chronic obstructive pulmonary disease (COPD) has been studied in many systematic reviews (SRs) and meta-analyses (MAs), but robust conclusions have not been reached due to wide variations in scopes, qualities, and outcomes. The aim of this overview was to determine the effectiveness of TIs on COPD patients.

**Methods:**

PubMed, EMBASE, Web of Science, and Cochrane Library were searched for all reviews on the topic of TI in treating COPD from inception to July 8, 2019, without restrictions on language. According to the inclusion and exclusion criteria, the retrieved literature studies were screened to select SRs and MAs of randomized control trials (RCTs) that evaluated the effects of TIs in COPD patients. The methodological quality of SRs and MAs was assessed with the AMSTAR-2 tool, and the strength of evidence was assessed with the grades of recommendations, assessment, development, and evaluation (GRADE) system for concerned outcomes in terms of mortality, quality of life (SGRQ total scores), exercise capacity (6MWD), and exacerbation-related outcomes (hospitalizations, exacerbation rate, and emergency room visits).

**Results:**

Our overview included eight SRs and MAs published in 2011 to 2019, from 95 RCTs involving 10632 participants. After strict evaluation by the AMSTAR-2 tool, 75% of the SRs and MAs in this overview had either low or critically low methodological quality. The effects of TIs for COPD on mortality, quality of life, exercise capacity, and exacerbation-related outcomes are limited, and all of these outcomes scored either low or very low quality of evidence on the GRADE system.

**Conclusions:**

There might be insufficient evidence to support the effectiveness of TIs for COPD currently, but the results of this overview should be interpreted dialectically and prudently, and the role of TIs in COPD needs further exploration.

## 1. Introduction

COPD is a complex chronic respiratory condition, usually caused by exposure to toxic gases or particles [1]. COPD is the leading cause of morbidity and mortality worldwide and carries a huge and growing economic and social burden [2, 3]. Its prevalence, morbidity, and mortality vary across countries and settings [1]. In China, COPD was the fourth leading cause of death in 2017 [4], affecting about 100 million patients nationwide, with the prevalence of about 13.7% for people over 40 years of age [5], which brought a huge social and economic burden [6]. Despite advanced medical, pharmacological, and scientific management, the patient's quality of life remains poor and exacerbation and mortality rates are still high [1].

In recent years, TIs (telemedicine, tele-healthcare, telerehabilitation, teleconsultation, telecare, telehealth, etc.) and self-management have played an increasingly important role in COPD, with the goal of improving clinical outcomes and reducing healthcare costs [7–10]. The latest document of Chronic Obstructive Pulmonary Disease [1] mentioned the role of TIs in COPD, but solely based on the results of a single study [11], the conclusions may not be objective enough.

At present, there have been a number of SRs and MAs on the effect of TIs for COPD. However, in most of those studies, it is shown that the effect of TIs for COPD is varied and heterogeneous, making it difficult to draw conclusions as for overall efficacy of TIs on COPD. The possible reason may be that SRs and MAs on the topic of TI for COPD vary in scopes, qualities, and outcomes, making the evidence difficult to conclude properly.

Therefore, we undertook an overview to evaluate the quality of the SRs and MAs and summarize the quality of evidence on their effects on mortality, quality of life, exercise capacity, and exacerbation-related outcomes, in order to identify the true efficacy and existing gaps in this area and to provide advices for future research.

## 2. Methods

This overview was conducted in accordance with the Preferred Reporting Items for Systematic Reviews and Meta-Analyses (PRISMA) guidelines [12] and the Methodology in Conducting a Systematic Review of Systematic Reviews of Healthcare Interventions [13]. No ethical application was required.

### 2.1. Literature Search

We systematically searched PubMed, EMBASE, Web of Science, and Cochrane Library for all reviews on the topic of TIs in treating COPD from inception to July 8, 2019, without restrictions on language. In PubMed, Cochrane Library, and Web of Science databases, we searched by combining the Medical Subject Headings (MeSH) and text words, while Emtree terms combined with free words were used for Embase database. In addition, the reference lists of all related reviews were also examined to ensure the comprehensiveness of the search. Two reviewers (XL Li and HL Zhao) performed the literature search independently, and any discrepancy was resolved with the experienced third reviewer (Y Xie). The retrieval strategies and steps were presented in Appendix 1 in Supplementary materials.

### 2.2. Inclusion and Exclusion Criteria

The reviews meeting all the following criteria were included: (1) performed in COPD patients with different grades of obstruction (GOLD I to IV) in stable periods; (2) assessing TIs (telemedicine, tele-healthcare, telerehabilitation, teleconsultation, telecare, telehealth, etc.) compared with a control group (usual care, ordinary health care, blank control, face-to-face support, etc.); and (3) reporting at least one of the following outcomes: mortality, hospitalizations, exacerbation rate, emergency room visits, and quality of life (SGRQ total scores). Exclusion criteria were as follows: (1) the comparison between two different TIs or the technique used in usual care; (2) reviews including random cluster studies.

### 2.3. Literature Selection

The eligible reviews were selected by two independent reviewers (HL Zhao and HL Zhang) in a two-step process. Firstly, abstracts of the identified reviews were screened for potential eligibility in the EndNoteX8 software after removing duplications. Then, the full texts were retrieved for further evaluation. A third coauthor (XQ Yu) resolved any arguments occurred as an arbiter.

### 2.4. Data Extraction

According to the characteristics of the included reviews, two researchers (Li and Xie) extracted the following basic information independently: the first author, publication year, country, searched databases, numbers of included trials and participants, quality assessment methods, interventions, and outcomes of each included reviews.

### 2.5. Quality Assessment

The AMSTAR-2 tool [14] was used to assess the quality of the included reviews, which contains 16 items, with 7 critical items. The situation of each item (especially the critical item) should be fully considered and categorized into 4 levels, namely, high, moderate, low, and critically low. AMSTAR-2 is not intended to generate an overall score. The quality of the included reviews was performed by two coauthors (Li and Zhang), and a third coauthor (Li) would involve as an arbiter if any arguments occurred.

### 2.6. GRADE Scoring

The GRADE system [15] was used to assess the evidence quality of concerned outcomes and classifies evidence quality into 4 levels: high, moderate, low, or very low. Evidence based on RCTs is regarded as high quality, but the credibility would be decreased if there are study limitations, inconsistency of results, indirectness of evidence, imprecision, and reporting bias. Two authors (Li and Yu) independently assessed the 5 items and resolved any ambiguities through discussing with the third coauthor (Li).

## 3. Results

### 3.1. Study Identification

**Figure 1:**
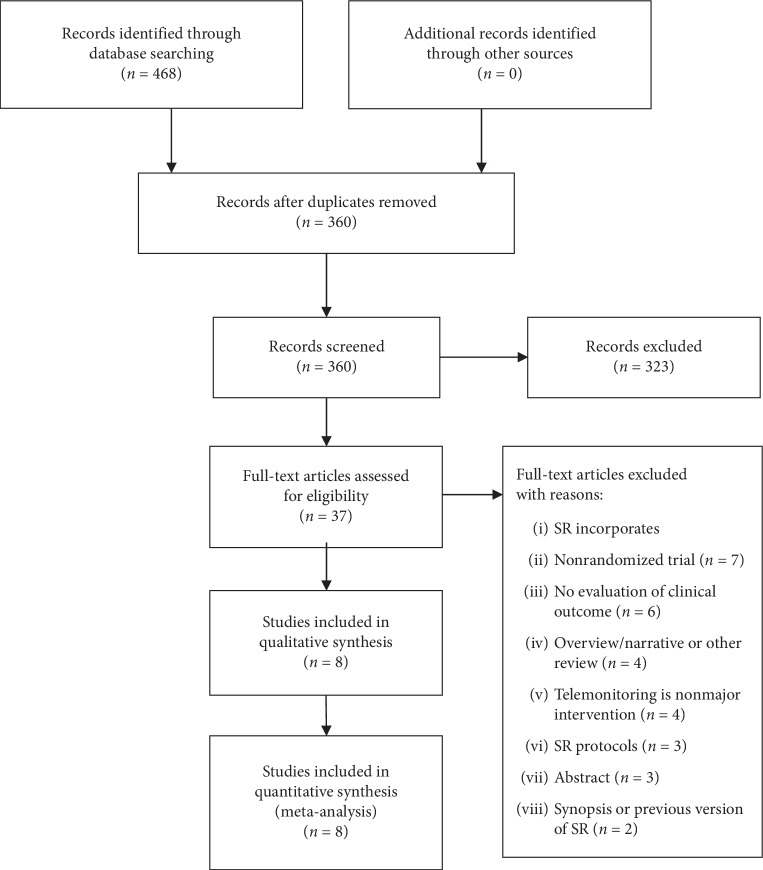
Study selection process for this overview.

In total, 478 literature studies were identified through database searching and 108 duplicated literature studies were excluded. After screening the titles and abstracts, 323 literature studies were excluded because of irrelevant topic. Therefore, 37 full-text review articles were selected for further evaluation. 29 articles were excluded for the following reasons: 7 incorporate nonrandomized trial; 6 do not evaluate clinical outcomes; 4 use telemonitoring as nonmajor intervention; 3 were protocols of review; 3 were meeting abstracts; 2 were synopsis or previous version of reviews; 4 were overview, narrative, or other type of review. The list of excluded literature studies and reasons of exclusion are displayed in Appendix 2 in Supplementary materials. Thus, a total of 8 SRs and MAs [16–23] were finally included in this overview. The study selection process is shown in Figure 1.

### 3.2. Characteristics of Included Review

We included six systematic review and meta-analysis [16–18, 20, 22, 23], one meta-analysis [19], and one systematic review, which were published between 2011 and 2019, with four from Asian countries [16–19] and four from European countries [20–23], and all databases reviews retrieved ranged from one to six, with PubMed, Embase, and Cochrane Library as the most widely searched databases. The number of RCTs included in each review ranged from 3 to 28, and the total participants ranged from 391 to 3645. In the included reviews, Cochrane criteria (the risk of bias from the Method Guidelines for Systematic Reviews in the Cochrane Review Group) were used to evaluate the quality of the included literature. The main characteristics of included reviews are shown in Table 1.

### 3.3. Quality of Included Reviews

According to AMSTAR-2 classification, two reviews were regarded as high quality [20, 23], two as low quality [19, 22], and four as critically low quality [16–18, 21]. The result of AMSTAR-2 score showed that the key factors affecting the quality of the reviews were item 3 (only 1 review [22] explained the selection of the study designs for inclusion), and item 7 (only 2 reviews [20, 23] provided a list of excluded studies and justified the exclusions). The quality of all included reviews is shown in Table 2.

### 3.4. Quality of Evidence in Concerned Outcomes (GRADE)

The evidence level of all concerned outcomes determined by the GRADE system was low or very low due to the study limitations within the original trials, inconsistency, imprecision, and the possibility of publication bias. The detail of GRADE evaluation is shown in Table 3.

#### 3.4.1. Mortality

Four reviews [16, 18, 21, 23] reported the effects of TIs on mortality, three of them [16, 18, 23] combined the data into meta-analysis, and one [21] performed a narrative synthesis of the available data from the original trials. The results of the four reviews showed that there was no statistically significant difference in the reduction of mortality between the TIs and the control group. According to the GRADE system, the quality of evidence for mortality reported in three MAs [16, 18, 23] was low, and the main reason for the downgrade was risk of bias.

#### 3.4.2. Hospitalization Rate

The hospitalization rate was reported in four reviews, which included three MAs [16, 20, 23] and one SR [21]. These MAs [16, 20, 23] show that TI is not superior to the control group in reducing the hospitalization rate. In the SR [21], most studies reported a positive effect of TIs on hospitalization for any cause. The quality of evidence for TIs to reduce hospitalization rate in patients of COPD is very low to low.

#### 3.4.3. Exacerbation Rate

Three reviews reported the effects of interventions on exacerbation rate, two reviews [18, 20] combined the data into meta-analysis, and one [23] performed a narrative synthesis. In the narrative synthesis [23], two primary RCTs [24, 25] reported that TIs had an advantage in reducing total exacerbations. However, the results of the other two MAs [18, 20] showed no difference in reducing exacerbations between the two groups. According to the GRADE system, the quality of evidence for exacerbation rate was low.

#### 3.4.4. Emergency Room Visits

Three reviews reported on emergency room visits, included two MAs [16, 23] and one SR [21]. The SR [21] reported that there might be a positive effect of TIs on emergency room visits, but only one original research reached statistical significance. Two MAs [16, 23] showed that TIs were superior to the control group in reducing emergency room visits. The quality of evidence is low.

#### 3.4.5. Quality of Life

Six reviews used the total scores of St George's Respiratory Questionnaire (SGRQ) to access quality of life in COPD patients. TI was associated with a clinically significant increase in quality of life in only one MA [20], while the results of the other five MAs and SR [16, 18, 19, 21, 23] were not statistically significant. The quality of evidence for quality of life was very low to low.

#### 3.4.6. Exercise Capacity

Two reviews [19, 22] used the 6-minute walk distance (6MWD) for exercise capacity of life in COPD patients. TI has neither statistical nor clinical significance in improving exercise capacity in two MAs [19, 22], and the quality of evidence for exercise capacity was very low to low.

Therefore, the impact of TIs for COPD on mortality, quality of life, exercise capacity, and exacerbation-related outcomes is limited.

## 4. Discussion

### 4.1. Main Findings

This overview of 8 SRs and MAs published in 2011 to 2019 provided the clinical evidence on the effectiveness of TIs in treating COPD from 95 RCTs that included 10632 participants. 75% of the included SRs and MAs were regarded as critically low to low quality according to the AMSTAR-2 evaluation, mainly due to failure to provide a list of excluded studies and justify the exclusion (critical domain 7) as well as the failure to explain the design of the selected studies. These may lead to selection bias and reduce the reliability of the results to some extent. We assessed the quality of evidence of effects of TIs on mortality, quality of life, exercise capacity, and exacerbation-related outcomes for COPD by the GRADE system. We obtained that the impact of TIs for COPD on mortality, quality of life, exercise capacity, and exacerbation-related outcomes is limited.

### 4.2. Interpretation to Limited Efficacy of TI

Mortality is an important endpoint outcome for COPD patient [1]. However, in our included MAs and SR, mortality is mostly a secondary outcome, and the results show that telemonitoring has a tendency to reduce mortality in patients with COPD, but it has not reached statistical significance. This result was similar to the findings of previous studies, which suggested that telemonitoring might fail in reducing mortality [11, 20, 26]. In most countries, COPD is one of the most important causes of death, but it was difficult to identify whether the cause of death was COPD-related or not. Thus, it was more likely to be completely removed from the death certificate, which may lead to poor reliability of mortality rate [27]. In addition, the included RCTs had a shorter follow-up period and varying severity of COPD patients, which might be insufficient for measurement of an effect on mortality.

SGRQ is a comprehensive disease-specific questionnaire for measuring the quality of life of the COPD patient [28], a decrease of 4 units in the SGRQ total scale means better quality of life in COPD [29]. The result of the total score of SGRQ is partly inconsistent in this overview; one MA [20] showed that TI improved quality of life assessed by SGRQ, and the change of total score of SGRQ was statistically significant, but not clinically significant. Another one MA [23] showed that the change of total score of SGRQ was clinically significant but not statistically significant. The possible explanation for this result is that the included SRs and MAs were different in inclusion and exclusion criteria as well as each TI differed among primary RCTs.

6MWD is a reliable, effective, and reactive measure of exercise capacity and an effective endpoint of patients with COPD [30]. In this overview, TI has neither statistical nor clinical significance in improving exercise capacity. According to the interpretation of the two MAs [19, 22], the difference in measurement methods and the characteristics of the 6-minute walk test may lead to underestimation of the reliability of the results.

Reducing the hospitalization rate, emergency room visits, and exacerbation rate is an important management objective of COPD [1]. Results of reducing the hospitalization rate reported in this overview are not significant, both in the dichotomous variables and continuous variables and in the subgroup analysis based on the severity of COPD and different interventions [16, 20, 23]. Exacerbation rate also showed similar result [18, 20, 23]. Results of emergency room visits are somewhat contradictory. Although the results of two MAs [16, 23] showed that TI can reduce the number of emergency room visits, their measurement standards are inconsistent, the effect value is small, and the quality of evidence is low. Our overview shows that TI in reducing the exacerbation-related rate was limited. Several potential reasons pertaining to this result should be mentioned. Firstly, the primary RCTs included were inconsistent in terms of exacerbation-related outcome measures. Studies of the definition and reporting of exacerbation events were varied from each other. Secondly, the duration of follow-up might be insufficient for longitudinal measurement of the efficacy. Lastly, COPD patients came from different settings and different severity, which affected the efficacy of TI to a certain extent.

### 4.3. Suggestions for Future Research

TI is a technology that captures data related to the diagnosis, prevention, and management of health and disease, making it possible to monitor and intervene in the event of acute and chronic disease [31], especially in the field of heart failure and COPD [10, 32]. But the impact on TIs for COPD on concerned outcomes is limited in this overview. Thus, clearer articulations are needed of how TIs can concretely affect clinical outcomes, along with more rigorous evaluations of clinical effectiveness [31]. We suggest that the primary RCTs for TI on COPD need to be designed with attention to detailed interventions and outcome measurements. Besides, longer follow-up research is needed to determine the long-term clinical effectiveness of TI. As for SR and MA, we suggest that subgroup analysis should be conducted strictly in accordance with consistent intervention, consistent treatment period and follow-up time, and consistent outcome measurement, so as to reduce bias when conditions permit.

### 4.4. Strengths and Limitations

To our knowledge, this overview is the first study to assess the methodological quality of SRs and MAs using the AMSTAR-2 tool and GRADE system to evaluate the quality of evidence for the efficacy of TIs for COPD. We conducted systematic and thorough searches and reasonable literature screening, without language restrictions, which may strongly reduce possible selection bias. Furthermore, we only included SR and MA of randomized trials and excluded reviews with nonrandomized controlled trials and observational studies to reduce the risk of mixed bias.

This overview also has some limitations. Firstly, evaluating methodological or evidence quality via the AMSTAR-2 tool or GRADE system, respectively, is a subjective process. Although included SR and MA have been evaluated independently by two researchers and examined by a third researcher, there may still be some bias. Secondly, we only focused on some concerned outcomes, which might fail to show the comprehensively curative effect of TIs on different stages and severity of COPD. Thirdly, this overview only assessed the quality of evidence for some aimed outcomes, which may not reflect the overall efficacy of TIs for COPD. Thus, we still need to interpret the results of this overview dialectically and prudently.

## 5. Conclusions

The current SR and MA revealed that TIs might not reduce mortality and improve quality of life, exercise capacity, and exacerbation-related outcomes in COPD patients. However, considering the limitations of our overview, more rigorous and scientific studies are needed in the future to further explore the efficacy of TIs in COPD.

## Figures and Tables

**Table 1:** Characteristics of the included reviews.

First author (years)	Databases searched	Country	No. of RCTs (no. of patients) included	Quality assessment for RCT/non-RCT	Intervention (technology)	Control	Outcomes	Study type
Youna et al, 2019 [16]	Ovid-Medline, Ovid-Embase, Cochrane Library	Korea	27 (3645)	Cochrane criteria	Telemonitoring (pulmonologist contact, telephone call, web-based exercise program)	Usual care (education self-care, clinical care, home exercises)	Mortality, emergency room visits, hospitalization, outpatient visits, length of stay, quality of life (SGRQ).	SR and MA

Yang et al, 2018 [17]	PubMed, Web of Science, Cochrane Library, Embase	China	6 (391)	Cochrane criteria	Mobile health applications (smartphones, networking/monitoring systems)	Usual care	Hospitalization, average days of hospitalization, exercise capacity, and activity levels	SR and MA

Ah-Ram et al, 2018 [18]	Medline, Embase, Cochrane Central Register of Controlled Trials, CINAHL	Korea	28 (2891)	Cochrane criteria	Telemonitoring (self-management and support program, telerehabilitation intervention, teleconsultation, telecare)	Usual care (face-to-face care or telephone consultation)	Mortality, exacerbation rate, quality of life (SGRQ)	SR and MA

Deng et al, 2018 [19]	PubMed, Embase, Web of Science, Cochrane Library	China	10 (1037)	Cochrane criteria	Telephone support (web-based call, phone calls, self-management education)	Usual care (education session, ordinary care)	Exercise capacity (6WMD), quality of life (SGRQ)	MA

McCabe et al, 2017 [20]	CENTRAL, Medline, Embase, CINAHL, AMED, PsycINFO	Ireland	3 (557)	Cochrane criteria	Web 2.0-based interventions (PCs, apps, iPad, Android tablets, smart phones, Skype)	Usual care (face-to-face/hard copy/digital documentary educational/self-management support)	Hospitalization, exacerbation rate, quality of life (SGRQ), self-efficacy (COPD self-efficacy Scale), cost-effectiveness, exercise capacity (6MWD), lung function (FEV_1_, FEV_1_% predicted) anxiety and depression, sustained behavior change	SR and MA

Pedone et al, 2015 [21]	PubMed	Italy	12 (1129)	Cochrane criteria	Telemonitoring (web-based call center, video conference, home-telephone line, touch screen equipment)	Not report	Mortality, hospitalization, emergency room visits, quality of life (SGRQ), patients' satisfaction	SR

Lundell et al, 2015 [22]	CENTRAL, PubMed, CINAHL, AMED, PsycINFO, Web of Science, Scopus, PEDro	Sweden	9 (982)	Cochrane criteria	Tele-healthcare (phone calls, web-based call, phone reminders, Skype)	Usual care (optimized medication, ordinary healthcare contacts)	Physical activity level, physical capacity (6MWD), dyspnea	SR and MA

McLean et al, 2011 [23]	CENTRAL, Medline, Embase, CINAHL, AMED, PsycINFO	UK	12 (1004)	Cochrane criteria	Tele-healthcare (telephones, video cameras, internet to communicate with a nurse or doctor)	Usual care (universal health program, advice face-to-face, education and home visits, standard home healthcare)	Mortality, exacerbation rate, quality of life (SGRQ), emergency room visits, hospitalization, lung function (FEV1, FVC), patient satisfaction, study withdrawal, cost, cost-effectiveness	SR and MA

SR: systematic review; MA: meta-analysis.

**Table 2:** Critical appraisal of included reviews through using the AMSTAR-2 tool.

No.	Item	[16]	[17]	[18]	[19]	[20]	[21]	[22]	[23]
1	Did the research questions and inclusion criteria for the review include the components of PICO?	1	1	1	1	1	1	1	1

2	Did the report of the review contain an explicit statement that the review methods were established prior to the conduct of the review and did the report justify any significant deviations from the protocol?^*∗*^	0.5	0	0	1	0.5	0	1	1

3	Did the review authors explain their selection of the study designs for inclusion in the review?	0	0	0	0	0	0	1	0

4	Did the review authors use a comprehensive literature search strategy?^*∗*^	1	1	1	1	1	0.5	1	1

5	Did the review authors perform study selection in duplicate?	1	1	1	1	1	1	1	1

6	Did the review authors perform data extraction in duplicate?	1	1	1	1	1	1	1	1

7	Did the review authors provide a list of excluded studies and justify the exclusions?^*∗*^	0	0	0	0	1	0	0	1

8	Did the review authors describe the included studies in adequate detail?	1	1	1	1	1	1	1	1

9	Did the review authors use a satisfactory technique for assessing the risk of bias (RoB) in individual studies that were included in the review?^*∗*^	1	1	1	1	1	1	1	1

10	Did the review authors report on the sources of funding for the studies included in the review?	1	0	1	1	1	0	1	1

11	If meta-analysis was performed, did the review authors use appropriate methods for statistical combination of results?^*∗*^	1	1	1	1	1	NP	1	1

12	If meta-analysis was performed, did the review authors assess the potential impact of RoB in individual studies on the results of the meta-analysis or other evidence synthesis?	1	1	1	1	1	NP	1	1

13	Did the review authors account for RoB in primary studies when interpreting/discussing the results of the review?^*∗*^	1	1	0	1	1	1	1	1

14	Did the review authors provide a satisfactory explanation for, and discussion of, any heterogeneity observed in the results of the review?	1	0	0	1	1	0	0	1

15	If they performed quantitative synthesis did the review authors carry out an adequate investigation of publication bias (small study bias) and discuss its likely impact on the results of the review?^*∗*^	1	1	1	1	1	NP	1	1

16	Did the review authors report any potential sources of conflict of interest, including any funding they received for conducting the review?	1	0	1	1	1	0	1	1

Overall quality		CL	CL	CL	L	H	CL	L	H

^*∗*^AMSTAR-2 critical domains; 1: yes; 0.5: partial yes; 0: no; NP: meta-analysis not performed; H: high; M: moderate; L: low; CL: critically low.

**Table 3:** Quality of evidence in included reviews with GRADE.

Outcome	Systematic review	N/n	Effect (95%)	GRADE					Quality of evidence
				Risk of bias	Inconsistency	Indirectness	Imprecision	Publication bias	
*Mortality outcomes*									
Mortality	Hong and Lee [16]	8 (1518)	RR 0.85 [0.64, 1.13]	–2	–1	0	0	0	L
	Sul et al. [18]	7 (919)	RR 0.89 [0.60, 1.34]	–1	–1	0	0	0	L
	McLean et al. [23]	3 (503)	RR 1.05 [0.63, 1.75]	–1	0	0	–1	0	L

*Exacerbation outcomes*									
Hospitalizations	Hong and Lee [16]	14 (2007)	RR 0.88 [0.80, 0.97]	–1	–1	0	0	0	L
	McCabe et al. [20]	1 (239)	OR 1.60 [0.80, 3.20]	–1	—	0	–1	0	L
	McLean et al. [23]	4 (604)	OR 0.46 [0.33, 0.65]	–1	0	0	0	0	M
Exacerbation rate	Sul et al. [18]	6 (NR)	RR 0.67 [0.31, 1.42]	–1	0	0	–1	0	L
	McCabe et al. [20]	1 (239)	OR 1.40 [0.70, 2.80]	–1	—	0	–1	0	L
Emergency room visits	Hong and Lee [16]	11 (1282)	RR 0.63 [0.55, 0.72]	–1	–1	0	0	0	L
	McLean et al. [23]	3 (449)	OR 0.27 [0.11, 0.66]	–1	0	0	–1	0	L

*Quality of life*									
SGRQ total scores	Hong and Lee [16]	4 (604)	MD –0.21 [–3.29, 2.86]	–1	0	0	–1	0	L
	Sul et al. [18]	9 (522)	MD 0.14 [–3.96, 4.23]	–1	–1	0	–1	0	VL
	Deng et al. [19]	6 (712)	SMD –0.36 [–0.51, 0.06]	–1	–1	0	0	0	L
	McCabe et al. [20]	3 (472)	MD –0.22 [–0.40, –0.03]	–1	–1	0	0	0	L
	McLean et al. [23]	2 (253)	MD –6.57 [–13.62, 0.48]	–1	–1	0	–1	0	VL

*Exercise capacity*									
6MWD	Deng et al. [19]	7 (570)	SMD 0.30 [0.00, 0.60]	–1	–1	0	0	0	L
	Lundell et al. [22]	5 (NR)	MD –1.3 [–8.10, 5.50]	–1	–1	0	–1	0	VL

NR: not reported; -2: very serious; -1: serious; 0: not serious; /: inapplicability; VL: very low; L: low; M: moderate.

## Supplementary Materials

Supplementary Materials Appendix 1: search strategy. The retrieval strategies and steps for searching PubMed, EMBASE, Web of Science, and Cochrane Library. Appendix 2: studies excluded. The list of excluded literature studies and reasons of exclusion were displayed in it. Appendix 3: PRISMA-checklist. PRISMA-checklist was used to normalize the report of this overview, in which the page numbers of the content were detailed.
