# Supplementary material for: Telemonitoring Interventions in COPD Patients: Overview of Systematic Reviews
Source: Biomed Res Int. 2020 Jan 16;2020:5040521. doi: 10.1155/2020/5040521 (PMC6988702; doi:10.1155/2020/5040521)
Supplement: Supplementary Materials — Appendix 1: search strategy. The retrieval strategies and steps for searching PubMed, EMBASE, Web of Science, and Cochrane Library. Appendix 2: studies excluded. The list of excluded literature studies and reasons of exclusion were displayed in it. Appendix 3: PRISMA-checklist. PRISMA-checklist was used to normalize the report of this overview, in which the page numbers of the content were detailed. [file 5040521.f1.zip › 5040521.f1/Appendix/Appendix 1_Search strategy.docx]

**Appendix Ⅰ**

**Details of the Literature Search Strategy**

(1) PubMed （1977 to July 8, 2019）

| **Search** | **Query** | **Items found** |
| --- | --- | --- |
| #1 | ("Pulmonary Disease, Chronic Obstructive"[Mesh]) OR "Lung Diseases, Obstructive"[Mesh] | 202976 |
| #2 | (((((((((((Pulmonary Disease, Chronic Obstructive[Title/Abstract]) OR Lung Diseases, Obstructive[Title/Abstract]) OR COPD[Title/Abstract]) OR Chronic Obstructive Pulmonary Disease[Title/Abstract]) OR COAD[Title/Abstract]) OR Chronic Obstructive Airway Disease[Title/Abstract]) OR Chronic Obstructive Lung Disease[Title/Abstract]) OR Airflow Obstruction*, Chronic[Title/Abstract]) OR Chronic Airflow Obstruction*[Title/Abstract]) OR Obstructive Lung Disease*[Title/Abstract]) OR Obstructive Pulmonary Disease*[Title/Abstract]) OR Pulmonary Disease*, Obstructive[Title/Abstract] | 54387 |
| #3 | #1OR#2 | 221574 |
| #4 | ((((("Telemedicine"[Mesh]) OR "Remote Consultation"[Mesh]) OR "Telerehabilitation"[Mesh]) OR "Telephone"[Mesh]) OR "Videoconferencing"[Mesh]) OR "Monitoring, Physiologic"[Mesh] | 207004 |
| #5 | (((((((((((((((((((((((((((((((((((((((((((((Mobile Health[Title/Abstract]) OR Health, Mobile[Title/Abstract]) OR mHealth[Title/Abstract]) OR m-Health[Title/Abstract]) OR Telehealth[Title/Abstract]) OR Tele-health[Title/Abstract]) OR eHealth[Title/Abstract]) OR e-Health[Title/Abstract]) OR Tele-medicine[Title/Abstract]) OR Tele medicine[Title/Abstract]) OR Teleconsultation*[Title/Abstract]) OR Tele-consultation[Title/Abstract]) OR Consultation, Remote[Title/Abstract]) OR Telerehabilitation*[Title/Abstract]) OR Tele-rehabilitation*[Title/Abstract]) OR Tele rehabilitation[Title/Abstract]) OR Remote Rehabilitation*[Title/Abstract]) OR Rehabilitation*, Remote[Title/Abstract]) OR Virtual Rehabilitation*[Title/Abstract]) OR Rehabilitation*, Virtual[Title/Abstract]) OR Telephone*[Title/Abstract]) OR Switchboard Service*[Title/Abstract]) OR Service*, Switchboard[Title/Abstract]) OR Smartphone[Title/Abstract]) OR Cell phone[Title/Abstract]) OR Microcomputer[Title/Abstract]) OR Videoconferenc*[Title/Abstract]) OR Teleconference[Title/Abstract]) OR Tele-communication[Title/Abstract]) OR Telecommunication[Title/Abstract]) OR Physiologic* Monitoring[Title/Abstract]) OR Monitoring, Physiological[Title/Abstract]) OR Patient Monitoring[Title/Abstract]) OR Monitoring, Patient[Title/Abstract]) OR Telenursing[Title/Abstract]) OR tele-monitoring[Title/Abstract]) OR telemonitor*[Title/Abstract]) OR home monitoring[Title/Abstract]) OR tele homecare[Title/Abstract]) OR tele-homecare[Title/Abstract]) OR tel care[Title/Abstract]) OR tele-care[Title/Abstract]) OR tele support*[Title/Abstract]) OR tele-support*[Title/Abstract]) OR tele manage*[Title/Abstract]) OR tele-manage*[Title/Abstract] | 127305 |
| #6 | #4 OR #5 | 283923 |
| #7 | #3 AND #6 | 3798 |
| #8 | Filters: Meta-Analysis; Systematic Reviews | 211 |

(2) Embase (1974 to July 8, 2019)

| **Search** | **Query** | **Items found** |
| --- | --- | --- |
| #1 | 'chronic obstructive lung disease'/exp | 122,856 |
| #2 | 'copd':ab,ti OR 'chronic obstructive pulmonary disease':ab,ti OR coad:ab,ti OR 'chronic obstructive airway disease':ab,ti OR 'chronic obstructive lung disease':ab,ti OR 'airflow obstruction*, chronic':ab,ti OR 'lung disease, obstructive':ab,ti OR 'obstructive lung disease*':ab,ti OR 'obstructive pulmonary disease*':ab,ti OR 'pulmonary disease*, obstructive':ab,ti | 113,279 |
| #3 | 'telenursing'/exp OR 'physiologic monitoring'/exp OR 'videoconferencing'/exp OR 'mobile phone'/exp OR 'telephone telemetry'/exp OR 'information service'/exp OR 'telephone interview'/exp OR 'telephone'/exp OR 'telerehabilitation'/exp OR 'teleconsultation'/exp OR 'telemedicine'/exp | 122,294 |
| #4 | 'mobile health':ab,ti OR 'health, mobile':ab,ti OR mhealth:ab,ti OR 'm health':ab,ti OR telehealth:ab,ti OR 'tele health':ab,ti OR ehealth:ab,ti OR 'e health':ab,ti OR 'tele medicine':ab,ti OR teleconsultation*:ab,ti OR 'tele consultation':ab,ti OR 'consultation, remote':ab,ti OR telerehabilitation*:ab,ti OR 'tele rehabilitation*':ab,ti OR 'tele rehabilitation':ab,ti OR 'remote rehabilitation*':ab,ti OR 'rehabilitation*, remote':ab,ti OR 'virtual rehabilitation*':ab,ti OR 'rehabilitation*, virtual':ab,ti OR telephone*:ab,ti OR 'switchboard service*':ab,ti OR 'service*, switchboard':ab,ti OR smartphone:ab,ti OR 'cell phone':ab,ti OR microcomputer:ab,ti OR videoconferenc*:ab,ti OR teleconference:ab,ti OR 'tele communication':ab,ti OR telecommunication:ab,ti OR 'physiologic* monitoring':ab,ti OR 'monitoring, physiological':ab,ti OR 'patient monitoring':ab,ti OR 'monitoring, patient':ab,ti | 119,605 |
| #5 | 'tele monitoring':ab,ti OR telemonitoring:ab,ti OR 'home monitoring':ab,ti OR 'tele homecare':ab,ti OR 'tel care':ab,ti OR 'tele care':ab,ti OR 'tele support*':ab,ti OR 'tele support':ab,ti OR 'tele manage*':ab,ti OR 'tele management':ab,ti | 4,526 |
| #6 | 'meta analysis'/exp OR 'meta analysis (topic)'/exp OR 'systematic review'/exp OR 'systematic review (topic)'/exp | 338,938 |
| #7 | 'meta-analysis as topic':ab,ti OR 'systematic reviews as topic':ab,ti OR 'meta analysis':ab,ti OR 'review, systematic':ab,ti OR 'systematic review':ab,ti | 265,839 |
| #8 | #1 OR #2 | 150,260 |
| #9 | #3 OR #4 OR #5 | 184,362 |
| #10 | #6 OR #7 | 396,546 |
| #11 | #8 AND #9 AND #10 | 112 |

(3) Cochrane Library (July 8, 2019)

| **Search** | **Query** | **Items found** |
| --- | --- | --- |
| #1 | MeSH descriptor: [Pulmonary Disease, Chronic Obstructive] explode all trees | 4813 |
| #2 | MeSH descriptor: [Lung Diseases, Obstructive] explode all trees | 17939 |
| #3 | (“Pulmonary Disease, Chronic Obstructive”):ti,ab,kw OR (“COPD”):ti,ab,kw OR (“Chronic Obstructive Pulmonary Disease”):ti,ab,kw OR (“COAD”):ti,ab,kw OR (“Chronic Obstructive Airway Disease”):ti,ab,kw | 17596 |
| #4 | (“Chronic Obstructive Lung Disease”):ti,ab,kw OR (“Airflow Obstruction*, Chronic”):ti,ab,kw OR (“Chronic Airflow Obstruction*”):ti,ab,kw OR (“ Lung Diseases, Obstructive”):ti,ab,kw OR (“Obstructive Lung Disease*”):ti,ab,kw | 8161 |
| #5 | (“Obstructive Pulmonary Disease*”):ti,ab,kw OR (“Pulmonary Disease*, Obstructive”):ti,ab,kw | 9965 |
| #6 | #1 OR #2 OR #3 OR #4 OR #5 | 30956 |
| #7 | MeSH descriptor: [Remote Consultation] explode all trees | 361 |
| #8 | MeSH descriptor: [Telemedicine] explode all trees | 2120 |
| #9 | MeSH descriptor: [Telerehabilitation] explode all trees | 77 |
| #10 | MeSH descriptor: [Telephone] explode all trees | 3030 |
| #11 | MeSH descriptor: [Videoconferencing] explode all trees | 181 |
| #12 | MeSH descriptor: [Monitoring, Physiologic] explode all trees | 11677 |
| #13 | MeSH descriptor: [Telenursing] explode all trees | 28 |
| #14 | #7 OR #8 OR #9 OR #10 OR #11 OR #12 OR #13 | 16078 |
| #15 | (“Mobile Health”):ti,ab,kw OR (“Health, Mobile”):ti,ab,kw OR (“mHealth”):ti,ab,kw OR (“m-Health”):ti,ab,kw  OR (“Telehealth”):ti,ab,kw | 2622 |
| #16 | (“Tele-health”):ti,ab,kw OR (“eHealth”):ti,ab,kw OR (“e-Health”):ti,ab,kw OR (“Tele-medicine”):ti,ab,kw OR (“Tele medicine”):ti,ab,kw | 906 |
| #17 | (“Teleconsultation*”):ti,ab,kw OR (“Tele-consultation”):ti,ab,kw OR (“Consultation, Remote”):ti,ab,kw OR (“Telerehabilitation*”):ti,ab,kw OR (“Tele-rehabilitation*”):ti,ab,kw | 1003 |
| #18 | (“Tele rehabilitation”):ti,ab,kw OR (“Remote Rehabilitation*”):ti,ab,kw OR (“Rehabilitation*, Remote”):ti,ab,kw OR (“Virtual Rehabilitation*”):ti,ab,kw OR (“Rehabilitation*，Virtual”):ti,ab,kw | 149 |
| #19 | (“Telephone*”):ti,ab,kw OR (“Switchboard Service*”):ti,ab,kw OR (“Rehabilitation*, Remote”):ti,ab,kw OR (“Smartphone”):ti,ab,kw OR (“Cell phone”):ti,ab,kw | 19143 |
| #20 | (“Microcomputer”):ti,ab,kw OR (“Videoconferenc*”):ti,ab,kw OR (“Teleconference”):ti,ab,kw OR (“Tele-communication”):ti,ab,kw OR (“Telecommunication”):ti,ab,kw | 721 |
| #21 | (“Physiologic* Monitoring”):ti,ab,kw OR (“Monitoring, Physiological”):ti,ab,kw OR (“Patient Monitoring”):ti,ab,kw OR (“Monitoring, Patient”):ti,ab,kw OR (“Telenursing”):ti,ab,kw | 3251 |
| #22 | (“tele-monitoring”):ti,ab,kw OR (“telemonitor*”):ti,ab,kw OR (“home monitoring”):ti,ab,kw OR (“tele homecare”):ti,ab,kw OR (“tele-homecare”):ti,ab,kw | 613 |
| #23 | (“tele-care”):ti,ab,kw OR (“tele support*”):ti,ab,kw OR (“tele-support*”):ti,ab,kw OR (“tele manage*”):ti,ab,kw OR (“tele-manage*”):ti,ab,kw | 34 |
| #24 | #15 OR #16 OR #17 OR #18 OR #19 OR #20 OR #21 OR #22 OR #23 | 26270 |
| #25 | #14 OR #24 | 38410 |
| #26 | #6 AND #25 | 1081 |
| #27 | MeSH descriptor: [Meta-Analysis as Topic] explode all trees | 291 |
| #28 | MeSH descriptor: [Systematic Reviews as Topic] explode all trees | 12 |
| #29 | (“Meta-Analysis”):ti,ab,kw OR (“Systematic Review*”):ti,ab,kw OR (“Review, Systematic”):ti,ab,kw | 28303 |
| #30 | #27 OR #28 OR #29 | 28308 |
| #31 | #26 AND #30 | 21 |
| #32 | Filters: Cochrane Reviews; Other Reviews | 10 |

(4) [Web of Science](https://apps.webofknowledge.com/home.do?SID=6BQQjiiMCVa9MgFvRpC) core collection (1986 to July 8, 2019)

| **Search** | **Query** | **Items found** |
| --- | --- | --- |
| #1 | Topic: (Pulmonary Disease, Chronic Obstructive) OR Topic: (COPD) OR Topic: (Chronic Obstructive Pulmonary Disease) OR Topic: (COAD) OR Topic: (Chronic Obstructive Airway Disease) OR Topic: (Chronic Obstructive Lung Disease) OR Topic: (Airflow Obstruction*, Chronic) OR Topic: (Chronic Airflow Obstruction*) OR Topic: (Lung Diseases, Obstructive) OR Topic: (Obstructive Lung Disease*) OR Topic: (Obstructive Pulmonary Disease*) OR Topic: (Pulmonary Disease*, Obstructive) | 76408 |
| #2 | Topic: (Telemedicine) OR Topic: (Mobile Health) OR Topic: (Health, Mobile) OR Topic: (mHealth) OR Topic: (m-Health) OR Topic: (Telehealth) OR Topic: (Tele-health) OR Topic: (eHealth) OR Topic: (e-Health) OR Topic: (Tele-medicine) OR Topic: (Tele medicine) OR Topic: (Remote Consultation) OR Topic: (Teleconsultation*) OR Topic: (Tele-consultation) OR Topic: (Consultation, Remote) OR Topic: (Telerehabilitation) OR Topic: (Telerehabilitation*) OR Topic: (Tele-rehabilitation*) OR Topic: (Tele rehabilitation) OR Topic: (Remote Rehabilitation*) OR Topic: (Rehabilitation*, Remote) OR Topic: (Virtual Rehabilitation*) OR Topic: (Rehabilitation*, Virtual) OR Topic: (Telephone) OR Topic: (Telephone*) | 96401 |
| #3 | Topic: (Switchboard Service*) OR Topic: (Service*, Switchboard) OR Topic: (Smartphone) OR Topic: (Cell phone) OR Topic: (Microcomputer) OR Topic: (Videoconferencing) OR Topic: (Videoconferenc*) OR Topic: (Teleconference) OR Topic: (Tele-communication) OR Topic: (Telecommunication) OR Topic: (Monitoring, Physiologic) OR Topic: (Physiologic* Monitoring) OR Topic: (Monitoring, Physiological) OR Topic: (Patient Monitoring) OR Topic: (Monitoring, Patient) OR Topic: (Telenursing) OR Topic: (tele-monitoring) OR Topic: (telemonitor*) OR Topic: (home monitoring) OR Topic: (tele homecare) OR Topic: (tele-homecare) OR Topic: (tele-care) OR Topic: (tele support*) OR Topic: (tele-support*) OR Topic: (tele manage*) | 286329 |
| #4 | #3 OR #2 | 367671 |
| #5 | #4 AND #1 | 3288 |
| #6 | Topic: (Meta-Analysis) OR Topic: (Systematic Review) OR Topic: (Review, Systematic) | 283241 |
| #7 | #6 AND #5 | 135 |
