# Supplementary material for: Telemonitoring Interventions in COPD Patients: Overview of Systematic Reviews
Source: Biomed Res Int. 2020 Jan 16;2020:5040521. doi: 10.1155/2020/5040521 (PMC6988702; doi:10.1155/2020/5040521)
Supplement: Supplementary Materials — Appendix 1: search strategy. The retrieval strategies and steps for searching PubMed, EMBASE, Web of Science, and Cochrane Library. Appendix 2: studies excluded. The list of excluded literature studies and reasons of exclusion were displayed in it. Appendix 3: PRISMA-checklist. PRISMA-checklist was used to normalize the report of this overview, in which the page numbers of the content were detailed. [file 5040521.f1.zip › 5040521.f1/Appendix/Appendix 2_Excluded studies.docx]

**Characteristics of excluded studies**

| **Author,Year** | **Tittle** | **Reasons** |
| --- | --- | --- |
| Gregersen TL,  2016 | Do telemedical interventions improve quality of life in patients with COPD? A systematic review. | SR incorporates nonrandomized trial |
| Alwashmi M,  2016 | The Effect of Smartphone Interventions on Patients With Chronic Obstructive Pulmonary Disease Exacerbations: A Systematic Review and Meta-Analysis. |  |
| Al Rajeh AM,  2016 | Monitoring of Physiological Parameters to Predict Exacerbations of Chronic Obstructive Pulmonary Disease (COPD): A Systematic Review. |  |
| Cruz J,  2013 | Monitoring of Physiological Parameters to Predict Exacerbations of Chronic Obstructive Pulmonary Disease (COPD): A Systematic Review. |  |
| Kamei T,  2013 | Systematic review and meta-analysis of studies involving telehome monitoring-based telenursing for patients with chronic obstructive pulmonary disease. |  |
| Bolton CE,  2011 | Insufficient evidence of benefit: a systematic review of home telemonitoring for COPD. |  |
| Polisena J,  2010 | Home telehealth for chronic obstructive pulmonary disease: a systematic review and meta-analysis. |  |
| Baroi S,  2018 | Advances in Remote Respiratory Assessments for People with Chronic Obstructive Pulmonary Disease: A Systematic Review. | No evaluation of clinical outcome |
| Martínez-García MDM, 2017 | Effectiveness of Smartphone Devices in Promoting Physical Activity and Exercise in Patients with Chronic Obstructive Pulmonary Disease: A Systematic Review. |  |
| Brunton L,  2015 | The Contradictions of Telehealth User Experience in Chronic Obstructive Pulmonary Disease (COPD): A Qualitative Meta-Synthesis. |  |
| Udsen FW,  2014 | A systematic review of the cost and cost-effectiveness of telehealth for patients suffering from chronic obstructive pulmonary disease. |  |
| Cruz J,  2014 | Home telemonitoring in COPD: A systematic review of methodologies and patients' adherence. |  |
| Bartoli L,  2009 | Systematic Review of Telemedicine Services for Patients Affected by Chronic Obstructive Pulmonary Disease (COPD). |  |
| Kruse C,  2019 | Telemonitoring to Manage Chronic Obstructive Pulmonary Disease: Systematic Literature Review. | Overview/Narrative or other review |
| Buekers J,  2018 | Oxygen saturation measurements in telemonitoring of patients with COPD: a systematic review. |  |
| Almojaibel AA.  2016 | Delivering Pulmonary Rehabilitation for Patients with Chronic Obstructive Pulmonary Disease at Home Using Telehealth: A Review of the Literature. |  |
| Franek J.  2012 | Home telehealth for patients with chronic obstructive pulmonary disease (COPD): An evidence-based analysis. |  |
| Qiu SH,  2018 | Using step counters to promote physical activity and exercise capacity in patients with chronic obstructive pulmonary disease: a meta-analysis. | Telemonitoring Interventions is non-major intervention |
| Turner BL,  2017 | Systematic review of the use of physical activity devices as an adjunct to pulmonary rehabilitation in patients with chronic obstructive pulmonary disease. |  |
| Wong CX,  2012 | Home care by outreach nursing for chronic obstructive pulmonary disease. The Cochrane database of systematic reviews. |  |
| Smith B,  2001 | Home care by outreach nursing for chronic obstructive pulmonary disease. |  |
| Janjua S,  2019 | Digital interventions for the management of chronic obstructive pulmonary disease. | SR protocal |
| Alghamdi SM,  2019 | Acceptance, adherence and dropout rates of individuals with COPD approached in telehealth interventions: a protocol for systematic review and meta-analysis. |  |
| Gaveikaite V,  2018 | Telehealth for patients with chronic obstructive pulmonary disease (COPD): a systematic review and meta-analysis protocol. |  |
| Sul AR,  2017 | Effectiveness of telemonitoring interventions for chronic obstructive pulmonary disease. | Abstract |
| Lundell S,  2014 | Telehealthcare for patients with COPD, effects on physical activity level, physical capacity and dyspnea: A systematic review and meta-analysis. |  |
| Jakobsen AS,  2012 | Varying effect of telemedicine in the treatment of chronic obstructive pulmonary disease--a systematic review |  |
| McLean S,  2012 | Telehealthcare for chronic obstructive pulmonary disease: Cochrane Review and meta-analysis. | Synopsis or previous version of SR |
| McLean S,  2009 | Telehealthcare for chronic obstructive pulmonary disease. |  |
